# Current Understanding of the Roles of Gut–Brain Axis in the Cognitive Deficits Caused by Perinatal Stress Exposure

**DOI:** 10.3390/cells12131735

**Published:** 2023-06-28

**Authors:** Mara Roxana Rubinstein, Adriana Laura Burgueño, Sofia Quiroga, Miriam Ruth Wald, Ana María Genaro

**Affiliations:** Laboratorio de Psiconeuroendocrinoinmunologia, Instituto de Investigaciones Biomédicas, Consejo Nacional de Investigaciones Científicas y Técnicas (CONICET)—Pontificia Universidad Católica Argentina, Buenos Aires C1107AFF, Argentina; alburgueno@conicet.gov.ar (A.L.B.); sofiaquiroga93@gmail.com (S.Q.); miriamruthwald@uca.edu.ar (M.R.W.)

**Keywords:** perinatal stress, cognitive deficit, gut–brain axis, environmental enrichment, melatonin

## Abstract

The term ‘perinatal environment’ refers to the period surrounding birth, which plays a crucial role in brain development. It has been suggested that dynamic communication between the neuro–immune system and gut microbiota is essential in maintaining adequate brain function. This interaction depends on the mother’s status during pregnancy and/or the newborn environment. Here, we show experimental and clinical evidence that indicates that the perinatal period is a critical window in which stress-induced immune activation and altered microbiota compositions produce lasting behavioral consequences, although a clear causative relationship has not yet been established. In addition, we discuss potential early treatments for preventing the deleterious effect of perinatal stress exposure. In this sense, early environmental enrichment exposure (including exercise) and melatonin use in the perinatal period could be valuable in improving the negative consequences of early adversities. The evidence presented in this review encourages the realization of studies investigating the beneficial role of melatonin administration and environmental enrichment exposure in mitigating cognitive alteration in offspring under perinatal stress exposure. On the other hand, direct evidence of microbiota restoration as the main mechanism behind the beneficial effects of this treatment has not been fully demonstrated and should be explored in future studies.

## 1. Introduction

The original epidemiological studies performed by David Barker in the early 1990s showed a link between restrictions in intrauterine growth and the frequency of cardiovascular diseases in adulthood. These findings led to a new field of research known as the developmental origins of health and diseases (DOHaD), asserting that the environment in the course of early life may influence the overall health status for a lifetime [1].

Several epidemiological studies on humans and experimental research on animals have demonstrated that an adverse perinatal environment has late negative consequences during life. These effects are mediated by epigenetic mechanisms, defined as heritable changes in gene expression that do not alter the underlying nucleotide sequence. These mechanisms include CpG methylation, chromatin remodeling, and regulatory noncoding RNAs [2].

Maternal health status is crucial in the development of the offspring, including physiological health and psychological functions. In the intrauterine stage, a fetus’ physiology is influenced by the psychological state of the pregnant mother. Pregnancy is a complex period full of changes, making women more vulnerable to stressful situations. Some authors refer to pregnancy as a critical period with high psychological sensitivity. The stress experienced by an individual during gestation is called prenatal stress (PS). During pregnancy, the mother’s endocrine, nervous, and immune systems adapt to promote a successful pregnancy. Dysregulation of the hypothalamic–pituitary–adrenal (HPA) axis has been a classical mechanism associated with the abnormalities induced by perinatal stress exposure (PNS). Physiological adaptive changes in pregnant women include a trend towards reduced stress responsiveness in the HPA axis and a switch in the immune system to favor an anti-inflammatory profile [3]. During the fetal period, the placenta forms a barrier against maternal glucocorticoids (GCs) through the activity of the glucocorticoid receptor and 11β-hydroxysteroid dehydrogenase (11β-HSD) type 2, the enzyme that converts GCs into inactive metabolites [4]. On the other hand, the enzyme 11β-HSD type 1 transforms inactive GCs to active GCs. Both enzymes provide an optimal cortisol level at the feto–maternal interface [5]. Stress-induced increases in maternal HPA axis activity may affect fetal HPA development and set lasting effects of stress during development [6].

Nowadays, there is a substantial body of evidence showing that PS is associated with detrimental health outcomes and neurobehavioral negative consequences in the offspring [7]. Animal studies have shown that PS impairs learning and memory and induces anxiety and depression-like behaviors in offspring. PS exposure has been described as causing stable, long-term changes to the central and peripheral stress response systems, increasing vulnerability to subsequent stress exposure in adulthood [8].

On the other hand, it was reported that the first thousand days of an infant’s life are essential for the child’s overall development and adult mental health. These periods are of significant vulnerability and may be influenced by internal and external risk factors [9].

Table 1 presents the articles, reviews, and meta-analyses performed in the last 10 years that show the principal findings on cognitive deficit caused by PNS in both experimental and clinical studies.

It is recognized that inflammation plays an important role in gene–environment interactions in neurodevelopmental disorders. The immune responses to environmental stimuli, such as stress in the perinatal period, can affect the neuro–immune signaling crucial to brain development [10,11].

The gut microbiota of both mother and infant is considered an important underlying contributor to fetal development. It is accepted that alterations in the commensal microorganisms in the body, especially during the first three years of life, can leave a lasting and potential footprint on health, contributing to the pathogenesis of multiple disorders [12]. Recently, the existence of a gut–brain axis was proposed as a bidirectional communication system that includes neural, immune, endocrine, and metabolic signaling [13].

In this review, we show experimental and clinical evidence of the role of the gut–brain axis in the early phase of brain development and, moreover, how PNS induces the disruption of this communication system, affecting cognitive performance in the offspring. Finally, we discuss potential early pharmacological and non-pharmacological treatments for preventing the deleterious effects of PNS. In this sense, melatonin use in the perinatal period and early enriched environment exposure could be valuable in reversing the negative consequences of early adversities.

**Table 1:** Overview of (a) articles, (b) meta-analyses, and (c) reviews from the last 10 years about perinatal stress effects and cognitive deficits in the offspring.

**(a)**						
**Type of Paper**	**Cohort**	**Article**		**Sex Affected**	**Mechanism**	
**Article**	**Animals**	Suenaga et al. (2012) [14]		Different effect in males and females	Changes in HP neuronal and glial markers	
		Adler and Schmauss (2016) [15]		Non-specified	↓ HDAC1 levels at promotors of distinct plasticity-associated genes	
		Wang et al. (2016) [16]		Females more affected than males	GluR expression changes within HP, PFC, and mammillary body	
		Reincke and Hanganu-Opatz (2017) [17]		Males more affected than females	Disturbed communication between PFC and HP	
		de Azeredo et al. (2017) [18]		Females more affected than males	Disruption of CDH adhesion function in HP	
		Zhang et al. (2017) [19]		Tested in males	↑ Autophagy level in the HP of male-offspring	
		Pascuan et al. (2017) [8]		Females	↓ BDNF, ↑ glucocorticoid receptors, and an alteration of Th1/Th2 in the HP	
		Goodwil et al. (2018) [20]		Females	↓ Expression and density of interneurons parvalbumin and in orbitofrontal cortex	
		Youssef et al. (2019) [21]		Both sexes	Cognitive deficits dependent on the estrous cycle phase in female	
		Chen et al. (2020) [22]		Both sexes	↑ Level of interleukin-18 in the dorsal and ventral HP	
		Li et al. (2020) [23]		Non-specified	Oxidative phosphorylation disorders in hippocampal neurons	
		Moura et al. (2020) [24]		Both sexes	Interfering with dentate gyrus assembly, affecting hippocampal function	
		Reshetnikov et al. (2020) [25]		Females	↓ The number of mature neurons in CA3	
		Kajimoto et al. (2021) [26]		Non-specified	↑ Hippocampal apoptotic response and downregulation of central serotonin pathway	
	**Human**	Laplante et al. (2018) [27]		Different effect in males and females	Non-specified	
		McQuaid et al. (2019) [28]		No significant sex-specific differences	↑ Gray matter density in bilateral PPC	
		Guo et al. (2020) [29]		No significant sex-specific differences	Non-specified	
		Cao-Lei et al. (2021) [30]		No significant sex-specific differences	Individual’s genotype alters their susceptibility to the effects of PS	
**(b)**						
**Type of paper**	**Cohort**	**Reference**	**N° of Studies Included**	**N° of Participants** **Included**	**Age**	**Principal Findings**
**Meta-analyses**	**Animals**	Bonapersona et al. (2019) [31]	212	8600 rodents	12 weeks	Promoted memory formation during stressful learning, but impaired non-stressful learning
		Rocha et al. (2021) [32]	45	451–763 rodents	>25 days	Decreased memory dependent on dorsal hippocampus
	**Human**	Tarabulsy et al. (2014) [33]	11	5903 mother–child dyads	0–60 months	Relative low relation between PS and child cognitive outcome
		Goodman et al. (2019) [34]	26	26,976 human adults	Non-specified	Exposure to early life stress associated with poorer working memory
		Delagneau et al. (2022) [7]	22	23,307 childrens	3 months–9 years	Weak negative association between PS and/or anxiety exposure and children’s general intellectual development
**(c)**						
**Type of Paper**	**Reference**		**Sex Affected**	**Mechanism**		
**Review**	Krugers and Joëls (2014) [35]		Non-specified	Alteration of the structure and function of the HP, amygdala, and PFC areas		
	Glover (2014) [36]		Non-specified	Increased exposure of the fetus to cortisol and serotonin, raised levels of inflammatory cytokines		
	Glover (2015) [37]		Non-specified	Non-specified		
	Hodes and Epperson (2019) [38]		Males	Lack of compensatory mechanisms and alterations in epigenetic regulation and organizational effects of hormones		
	Abbink et al. (2019) [39]		Non-specified	Astrocyte dysfunction		
	Lautarescu et al. (2019) [40]		Non-specified	Cortical thinning and an enlarged amygdala		
	Van den Bergh (2020) [41]		Non-specified	Aberrations in neurodevelopment, functional and structural brain connectivity, changes in HPA axis and autonomous nervous system		

Abbreviations used: ↓: decrease, ↑: increase, PPC: posterior parietal cortex, BDNF: brain-derived neurotrophic factor, Th: T helper, CA3: Cornu Ammonis 3, HDAC: histone deacetylase, GluR: glutamate receptor, HP: hippocampus, PFC: prefrontal cortex, CDH: cadherin. HPA: hypothalamic–pituitary–adrenal axis. PS: prenatal stress.

## 2. Gut–Brain Axis Role in Physiological and Pathological States

Microbiota is the group of microorganisms (commensal, symbiotic, and pathogenic) that are present in a defined environment [42]. Intestinal microbiota is involved in many processes, such as vitamin and nutrient synthesis, fiber digestion, and intestinal epithelial and mucosal barrier maintenance; additionally, it intervenes in intestinal defense against pathogens and modulates the immune system, participating in its development and regulation of immune responses [43]. Alternatively, a disrupted gut microbiota (dysbiosis) is associated with many diseases, including irritable bowel syndrome, allergies, cardiovascular disease, obesity, diabetes, and neurodevelopmental, behavioral and cognitive disorders [44]. An increase in intestinal permeability has been shown in animals with dysbiosis, leading to a rise in endotoxin circulation and immune activation [45].

Microbial exposure starts at birth or even in utero, but it is modified by factors such as diet, age, host genetics, antibiotic use, lifestyle, and environmental factors [46]. Highly variable microbial compositions and diversity have been demonstrated through sequencing studies across populations. Nevertheless, the main microbial components have been identified, but the relative proportions and species present may vary from one individual to another [47,48].

The adult gut microbiota is composed mainly of the phyla Firmicutes, Bacteroidetes, Proteobacteria, Actinobacteria, Verrucomicrobia, and Fusobacteria. Between 70 and 90% of the total abundance is composed of the phyla Firmicutes and Bacteroidetes. Prominent genera from Bacteroidetes phylum is represented by *Bacteroides*, *Parabacteroides*, and *Prevotella*. In the phylum Firmicutes, *Clostridium*, *Lactobacillus*, *Streptococcus*, *Enterococcus*, *Eubacterium*, and *Ruminococcus*, among others, are included. The phylum Actinobacteria accounts for less than 10% of the total gut microbiota, and its prominent genera are *Bifidobacterium* and *Collinsella*. The Proteobacteria phylum constitutes less than 2% of total abundance, and *Helicobacter* and *Escherichia* are the principal genera. Phyla Fusobacteria and Verrucomicrobia constitute less than 3% of the total gut microbial diversity and are dominated by the *Fusobacterium* and *Akkermansia* genera, respectively [49]. In general, it is accepted that *Bifidobacterium* and *Lactobacillus* strains provide benefits to the host and are commonly used as probiotics [50]. Recently, new beneficial gut bacterial species have been identified, including *Faecalibacterium prausnitzii*, *Ruminococcus bromii,* and *Akkermansia muciniphila* [50]. *Faecalibacterium prausnitzii* has anti-inflammatory properties and is one of the main producers of butyrate, *Ruminococcus bromii* is a fundamental species for degrading resistant starch that allows other bacteria to utilize the breakdown products, and *Akkermansia muciniphila* is a mucin-degrading bacterium providing oligosaccharides from mucin to other bacteria and produces acetate and propionate, which butyrate producers use. Its decrease has been associated with obesity and other metabolic diseases. On the other hand, pathobionts are opportunistic bacteria species with the potential to turn pathogenic under adverse conditions [51]. Their expansion occurs when there is an imbalance in the microbiota. Examples of pathobionts include *Clostridioides difficile*, *Helicobacter hepaticus*, *Helicobacter pylori*, segmented filamentous bacteria (SFB), invasive *Escherichia coli*, *Proteus mirabilis*, *Klebsiella pneumoniae*, *Prevotellaceae,* TM7, and vancomycin-resistant *Enterococcus* spp [51]. The beneficial use of prebiotics (nutrients that are degraded by gut microbiota, stimulating the growth of limited bacterial species) and probiotics (live microorganisms that provide a benefit on the host) to restore microbial composition has been known for many years. In general, probiotic intervention studies have used strains of *bifidobacteria* and *lactobacilli*, as they are recognized as safe, and their administration has shown effectiveness against many diseases, such as obesity, insulin resistance syndrome, type 2 diabetes, and non-alcoholic fatty liver disease, among others [52].

Using germ-free animals, it was discovered that the gut microbiota participates in the development, stabilization, and maturation of the immune system by shaping the immune tolerance and promoting the differentiation of immune cells [53].

**Figure 1:**
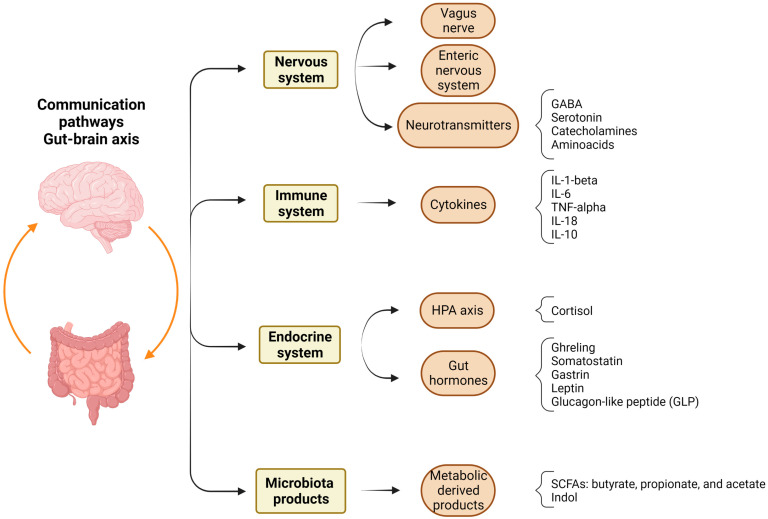
Cross-talk of the gut–brain axis. Different pathways participate in the bidirectional communication modulating gut–brain homeostasis. Gut dysbiosis affects neuropsychiatric health by inducing alterations in the signaling pathways of the gut–brain axis. GABA: gamma-aminobutyric acid, IL: interleukin, TNF: tumor necrosis factor, HPA: hypothalamic–pituitary–adrenal axis. SCFAs: short-chain fatty acids. Figure was created with the BioRender.com.

The gut–brain axis is defined as the bidirectional communication between the central and enteric nervous systems. It consists of the autonomic nervous system (including the vagus nerve), the HPA axis, the endocrine system, the immune system, and bacterial products and metabolites [54]. Figure 1 shows the communication pathways in the gut–brain axis. It has been shown that the gut microbiota contributes to neurodevelopment; germ-free mice show a decrease in anxiety-like behavior [55,56,57], reduced social behavior [58,59], memory deficits [60], and hyperreactivity on the HPA axis after restraint stress [61]. Furthermore, the production of neuroactive molecules can be modulated by gut microorganisms in both humans and mice [54]. The *Lactobacillus*, *Bifidobacteria*, *Enterococcus*, and *Streptococcus* species influence serotonin, GABA, and acetylcholine, thus affecting brain physiology [54]. Moreover, microbial metabolites from dietary tryptophan, a serotonin precursor, can act on microglia activation and astrocytes, modulating neuroinflammation [62]. Fermentation of dietary fibers by microbiota produces short-chain fatty acids (SCFAs): butyrate, propionate, and acetate. These products promote gut health by improving the integrity of the mucosal barrier and mucus production and reducing inflammation. They can cross the blood–brain barrier and participate in maintaining its integrity, preserving brain homeostasis [43,63,64].

In addition, many studies have demonstrated that stress can modulate microbiota compositions and reduce the richness and diversity of the gut microbiota [65,66,67]. Even a single 2 h exposure to a social stressor significantly changed the microbial profile and reduced the proportion of the main phyla in mice [68].

Perinatal maternal health significantly affects the offspring’s development. Moreover, it has been shown that PNS alters maternal microbiota and can be transmitted to the progeny [69]. The initial community that colonizes a newborn is provided by maternal vaginal and gut microbiota. Thus, any disturbance in maternal microbiota is transmitted to the offspring, disturbing their neuro–immune development and eliciting cognitive impairment and mood disorders, among other adverse health outcomes, in the progeny [69]. Accordingly, the mode of delivery (vaginal delivery or cesarean delivery) is fundamental, but at four months postpartum, the microbial community in the infant’s gut is replaced by microbial strains more similar to the maternal gut microbiota [70,71].

### 2.1. Perinatal Stress and Gut–Brain Axis

There has been an explosion of studies describing alterations in microbial compositions at different life periods after PNS in both animals and humans. A dysbiotic microbiota has been described in pregnant mice after stress exposure [72].

#### 2.1.1. Animal Studies

Exposure of pregnant mice to chronic variable stress during gestational days one to seven led to changes in fecal microbiota, showing a different shift in the microbial community structure between early and late pregnancy in mice exposed to stress [73]. Regarding the vaginal microbiota, microbial structures and compositions were altered in stressed dams, enabling the transmission of the altered communities during the delivery of the newborns [73]. The vaginal abundance of *Lactobacillus* was reduced after PS, decreasing this bacteria in the offspring [74]. The authors proposed that early PS may influence offspring development through alterations in gut microbial composition during pregnancy and the transmission of a dysbiotic vaginal microbiome at birth. Furthermore, a distinctive colonic and plasma metabolome was found in PS pups vs. control [74]. To study the direct role of maternal microbiota on offspring, the authors delivered control and PS pups by C-section and colonized the newborns with microbiota from either control or stress-exposed mothers [75]. The results showed a similar phenotype (altered microbiota compositions, changes in body weight, and increased corticosterone responses to an acute stressor) in the control offspring transplanted with vaginal microbiota from stress-exposed dams and in stress-exposed offspring. In contrast, PS offspring transplanted with vaginal microbiota from the control dams did not totally rescue the PS-exposed phenotype [75]. Thus, the effects of PS on offspring are not only a result of altered vaginal and gut microbiota, but instead a complex interplay between various factors, such as the impact of maternal antibodies or metabolites on the fetal gut [75].

In another model, C57BL/6 pregnant mice were exposed to 2 h-a day restraint stress between embryonic days 10 and 16. Microbial stool communities differed significantly between the stressed and control dams [76]. In the placenta, despite a low bacterial load, the sequences in the principal component analysis plotted differently between stressed and non-stressed dams. Interleukin (IL)-1β levels were elevated and the brain-derived neurotrophic factor (BDNF) was decreased in the placentas of stressed dams [76]. Intriguingly, different results were found in male and female offspring. Increased IL-1β was found in the fetal brains of PS female mice, while no changes were detected in the adult amygdalae. For BDNF, no significant changes were found in the fetal brain, but a decrease in the adult amygdala was observed in females born from stressed dams. These results were accompanied by cognitive impairment [76]. Regarding the gut microbiota, adult females showed differences in the overall microbial community and in the relative abundance of the main phyla, Bacteroidetes and Firmicutes, between the PS and control offspring [76]. Males born from stressed mothers did not show cognitive impairment, but they showed a reduction in social behavior. Increased IL-1β and IL-6 levels were found in the cortex, and a different gut microbial community with differences in the relative abundance of bacterial taxa were found in the PS males [77]. The alterations described in these models showed an intricate system where PS exposure leads to alteration in cognition that could be mediated through modifications in microbiota and inflammation. This was confirmed using C-C motif chemokine ligand (CCL2)^−/−^ and germ-free mice [78]. Germ-free mice exposed to PS were unable to induce placental and fetal brain inflammation (no increase in the chemokine CCL2 and IL-6). Thus, the immune process that occurs in utero after PS exposure is mediated by maternal microbes. CCL2^−/−^ mice exposed to PS failed to exhibit an increased IL-6 in the fetal brain, proving a complex interaction between the maternal microbiota and inflammation [78].

PS has long-term effects on an offspring’s gut microbiota. C57BL/6 mice were subjected to restrain stress from embryonic days 0.5 to 19.5, and their fecal microbiota was measured in 8-week-old offspring, showing alterations in richness, diversity, and community structure [79].

In rats exposed to restrain stress from embryonic days 14 to 20, there was no difference in microbial diversity or in relative abundance at the phyla level at four months of age [80]. Nevertheless, PS rats had a higher abundance in some families in the Clostridiales order and a reduced abundance in some of the Lactobacillales order. These animals also showed a hyperactive HPA axis response to stress, increased locomotor activity, and impaired cognitive function [80]. However, using a model of chronic, unpredictable mild stress during the 21 days of gestation in rats, a decrease in richness and diversity in maternal and offspring gut microbiota was found [81]. Additionally, there was a reduction in the abundance of *Lactobacillaceae* and an increase in *Muribaculaceae* in PS offspring. In parallel, changes in hippocampal structure and a decreased expression and signaling of BDNF/CREB were observed [81].

Regarding probiotic use, there are a few preclinical studies administrating probiotics to the mother and/or the offspring in the context of PNS exposure. These studies showed a reversal of behavioral deficits provoked by PNS exposure [82,83,84,85].

#### 2.1.2. Clinical Evidence

Many authors have assessed the effects of stress during pregnancy on adverse outcomes [86]. High levels of perceived stress or high cortisol levels were associated with intrauterine growth restriction, low gestational age and anthropometric measures, and poor infant neurodevelopment [86]. Interestingly, some studies have been performed on the association between PS and gut microbiota in humans.

A project, part of the Finn Brain Birth Cohort study, was developed to study the role of early life exposure on infant fecal microbiota [87]. For this project, 398 mothers were included, and an infant stool sample was taken at 2.5 months of age. The mothers completed questionnaires to evaluate maternal psychological stress during gestation. In addition, hair cortisol levels were measured at gestational week 24. Positive associations were found between maternal PS and bacterial genera from Proteobacteria phylum in infants, and a negative association was found with *Akkermansia*. In contrast, hair cortisol levels were negatively associated with *Lactobacillus*. There was no association with microbial diversity [87]. In a Dutch cohort of 56 vaginally born infants followed for 110 days after birth to study the development of the infant intestinal microbiota [88], participants were divided into low maternal stress and high maternal stress categories according to the scoring in the questionnaire responses of the mothers and their salivary cortisol measured at week 37 of pregnancy. A high relative abundance of the Proteobacteria group and low relative abundance of lactic acid bacteria and bifidobacteria were found in infants of mothers with high stress. The change in microbial compositions was related to increased gastrointestinal symptoms and allergic reactions reported by the mothers [88]. As part of the Healthy Babies Before Birth (HB3) longitudinal study, 46 pregnant women from the USA were enrolled and interviewed. Blood samples and medical records were collected at early, mid, and late pregnancy and at 4–8 weeks, 5–7 months, and 11–13 months postpartum [89]. Infant stool samples were taken at the postpartum visits. High anxiety and stress reported by the mothers were associated with reduced alpha diversity indices at 5–7 and 11–13 months postpartum. The taxonomic analysis revealed a positive association between low anxiety and low perceived stress with *Bifidobacterium dentium*, *Bifidobacterium longum*, and *Lactobacillus rhamnosus*, known as beneficial microbes. Additionally, prenatal maternal cytokines IL-6, IL-8, and IL-10 and the tumor necrosis factor (TNF)-α were associated with *Bifidobacterium dentium*, *Bifidobacterium longum*, *Lactobacillus rhamnosus*, and *Akkermansia muciniphila* [89]. These results showed that PS was associated with changes in the infant microbial community that may affect offspring health, indicating which maternal factors may be involved.

A similar study was conducted on Galápagos’ San Cristóbal island, Ecuador, on 25 pregnant women [90]. The women completed surveys about food insecurity, social support, depression, and stress, and salivary samples were taken for cortisol measurement during and after pregnancy. Infant stool samples were collected at two months of age. Results showed an association between maternal depression, stress, and high cortisol levels and a lower Shannon diversity index. Additionally, differences in beta diversity indices were found when comparing low and high levels of maternal and infant cortisol [90]. In addition, there is an added relevance to this study that lies in the sample’s origins: most microbiota research is performed in industrialized countries from North America, Europe, and China [91]; thus, few data come from South America as in this particular study.

In a very interesting longitudinal study [92], fecal samples from 89 one-year-old infants were collected and used for microbial analysis. Cognitive testing was performed in the participants at one and two years of age. A microbial cluster analysis identified three groups of infants that differed significantly at the cognitive testing at age two. In addition, a negative correlation was found between alpha diversity and cognitive performance (a higher alpha diversity was associated with a lower cognitive performance) [92]. Although this was a longitudinal study and no causal role can be attributed to this correlation, it shows an association between microbial composition and cognitive performance in infants.

There have been a few trials studying the effects of probiotic supplementation on prenatal maternal anxiety and depression. However, conflicting results were found; in some, there was no difference between placebo and probiotic treatment [93,94], and in others, a positive reduction in anxiety and depression was found [95]. Moreover, these studies did not consider the effects of supplementation in children.

The evidence described above affirms the importance of the gut–brain axis interaction in development and health. Psychological stress could be a factor that alters the dialogue between gut and brain and may lead to cognitive impairment, among other disorders. Future studies should evaluate the causality relations among PNS, microbial alterations, and offspring development.

## 3. Environmental Enrichment

Environmental enrichment (EE) is defined as the environment causing brain stimulation as a result of physical or social elements. EE, through higher physical, sensory, cognitive, and social stimulation, induces anatomical and molecular changes within the brain, resulting in significant improvements in sensorimotor and cognitive function in animal models of disease [96].

Donald Hebb [97] was the first to describe the relationship between EE and cognition and behavior. Hebb noted that the animals he had bought for his children, which were free, performed better on subsequent behavioral tasks than the rats accommodated in the laboratory cages. Then, there were a variety of variables, such as species, age, and sex, contributing to different results [98].

Moreover, it was shown that EE induces changes in brain neurochemistry and physiology. Several aspects of hippocampal function–such as neurogenesis, long-term potentiation and dendritic spine growth, neurotrophin mRNA expression, and the activation of mitogen-activated protein kinase (MAPK)–as well as cyclic adenosine monophosphate (cAMP) and its response element-binding protein (CREB) are increased after EE exposure [99].

On the neuronal level, EE has increased the size of neuronal cell bodies and nuclei, the number and size of dendrites and dendritic branching, and the number of dendritic spines [100,101]. Additionally, EE has altered glial cells in the brain [102,103].

Rosenzweig et al. [104] described in animals for the first time that EE incremented the activity of acetylcholinesterase (AChE), suggesting that EE impacts the cholinergic system. Posterior studies have supported this observation and extended it to other neurotransmitter systems with diffuse projections to the entire brain, such as the serotonergic and norepinephrine systems [105].

### 3.1. Animal Studies

EE has been proposed as a treatment for enhancing cognitive performance in rodents [106,107]. It is accepted that residing in EE conditions gives animals an optimal state, leading to an improvement in their cognitive activities and enhancing exploration, social interaction, and physical exercise [108]. This practice induces a positive neurobiological change [109]. Thus, it has been shown that EE improved behavioral, cellular, and molecular alterations in animal models of aging and neurological and mental disorders [108]. In Table 2, the beneficial effects of EE exposure in animal models of PNS are shown.

As mentioned, a relationship between early-life stress and cognitive deficits in animals and humans is widely documented. High cortisol levels, as induced by stress, have led to reduced neuronal survival, neuronal processes, and neurogenesis in brain regions that express a high density of glucocorticoid receptors, such as the hippocampus [110]. EE exposure in adolescent rats has been described as abolishing the deleterious effect induced by PS. Indeed, the animals exhibited improved social play behavior, the regularization of several circadian rhythms, and decreased anxiety and HPA axis reactivity [111].

Maternal separation (MS) during the postnatal period is another model of PNS that results in a greater response to stressors in adult life and the development of stress-related disorders, including anxiety and depression. MS has been reported to induce social and memory deficits in adult rodents. It has been shown that MS results in long-lasting HPA axis hyperactivity and memory impairment. These results were associated with an increase in arginine vasopressin expression due to DNA hypomethylation of the promoter region of this gene [112]. Adaptation to stress hormones shapes the glutamatergic response and hippocampal synaptic plasticity, thus modulating cognitive function [113]. In the MS model, EE increased the expression of BDNF, promoting the growth and maturation of neurons. It was demonstrated for MS that EE “rescued” neural plasticity and decreased anxiety by normalizing the structural enlargement of the basolateral amygdala [114]. EE appears to act as a developmental enhancer for balancing a previous lack of inputs or for boosting “normal” development.

EE has been described as increasing the trafficking of glutamate receptor subunits to the postsynaptic membrane in neurons of the hippocampus and other brain regions [115,116]. A possible role for the hippocampal corticotropin-releasing hormone (CRH) was suggested [117] in glutamatergic synaptic dysfunction and memory impairment in MS rats. When offspring exposed to MS were treated with a CRH type 1 receptor blocker, the authors observed that the escape latency time decreased, and the time spent in the target quadrant increased in the Morris water maze test. These findings suggest that regulation of CRH signaling via the type 1 receptor mediates hippocampal glutamatergic synaptic dysfunction induced by MS and memory impairment in rats. Furthermore, it was observed that histone hyperacetylation and DNA hypomethylation might be responsible for the increased hippocampal Crh expression in MS rats. The authors found similar cognitive improvements to those obtained by blocking the CRH type 1 receptor when exposing MS rats to EE. Thus, they suggested that EE might mitigate the hippocampal glutamatergic synaptic dysfunction and memory impairment induced by MS through the epigenetic suppression of Crh [118].

Gamma-aminobutyric acid (GABA), the primary inhibitory neurotransmitter in the brain, plays a master role in learning, memory [119], and synaptic plasticity [120]. In particular, GABAA receptors play a critical role in memory performance [121]. The hippocampus possesses numerous types of GABAergic neurons [122]. It has been reported that the administration of a GABAA receptor antagonist can enhance memory consolidation [123]. In addition, stress and corticosterone increased GABAergic transmission [124] and GABAA receptor expression [125] in the hippocampus.

It has been reported that EE reduced GABAergic inhibition by compensating for cognitive deficits and increasing synaptic plasticity in a murine model of Down syndrome [126]. Furthermore, it has also been reported that EE might reduce anxiety-related behaviors through its action on the GABAergic inhibitory system [127].

Recently, using a model of prenatal noise stress [128], it was reported that GABAergic agonist administration significantly decreased the effects of EE on spatial learning in stressed animals. Furthermore, EE and GABAergic antagonist administration individually enhanced hippocampal-dependent cognitive function. The authors proposed that there might be a cross interaction between EE and the suppression of GABAergic neurotransmission, as both improved hippocampal-dependent cognitive function.

It has been shown that GABAA antagonist administration can improve memory by increasing BDNF expression in the hippocampus [123,129]. Through changes in postsynaptic GABAA receptor expression and tonic GABAergic inhibition, corticosterone has also been reported to alter GABAergic signaling [130].

BDNF is critical in synapse formation, neuronal survival, and neuronal growth. It appears to be involved in mediating the effects of environmental manipulation on brain functioning during early life stages [131]. Furthermore, BDNF expression is sensitive to adverse life experiences [132,133]. It has been proposed that the BDNF profile of enriched animals may represent the neurobiological correlate of their resilience phenotype in a stressful situation [134].

Decreased BDNF levels have been reported in several brain regions of adult rats exposed to MS [135,136,137,138,139]. In addition, an increase in BDNF expression levels induced by EE was found in mice exposed to MS, restoring spatial memory deficits. This suggests that an increase in BDNF may be a prerequisite for the observed behavioral effects [139]. However, BDNF levels have been found to increase, decrease, or stay the same depending on the brain area under study [118,140], the duration of MS exposure, and the developmental period of that exposure. These findings show the complexity of the mechanisms affecting synaptic plasticity in the face of an adverse early life event.

Finally, EE improved spatial memory and prevented the degradation of attention performance in aged rats [141]. Moreover, EE was shown to increase the ability to acquire and use spatial information and to promote neurogenesis restoration in aged rats, probably as a result of the increased survival of neurons in the basalo–cortical system [142].

In a recent meta-analysis evaluating the effects of EE and stress on learning and memory in rodents, EE ameliorated the detrimental effects of stress on learning and memory. Moreover, there was a significant synergistic interaction between EE and stress, with EE providing a significantly greater benefit in stressed individuals than in individuals not exposed to stress manipulation [143].

The evidence mentioned above confirms the positive influence of EE exposure and a clear relation between early life experience and later brain structure and function. The relationship between childhood experience and normal brain development in humans is less documented. However, it has been shown that social environments that are extremely enriched or adverse can influence hippocampal volume [144].

### 3.2. Clinical Evidence

Clinical studies have shown that stimulation from both surrounding sources and EE exert a direct impact on the structural networks of the human brain [145,146]. In humans, it is impossible to control for all factors that might be considered as EE. Several authors have shown that physical exercise can produce some of the beneficial effects of EE on the brain and behavior [147]. Moreover, humans tend to live in environments rich in sensory stimuli, and where a person lives may be associated with differences in brain structure. A positive association has been observed between living near forests and amygdala integrity, suggesting that geographical characteristics may play an essential role in environmental enrichment [148]. In children, it was reported that, despite improving access to education worldwide, there is still an association between a child’s socio–economic status and their scores on performance tests and school grades [149]. Differences have also been observed in the brain structures of children and adolescents from families with different income levels, particularly in brain regions related to language, reading, executive function, and spatial skills [150,151]. It was suggested that, from an early age, parents’ socio-economic status could influence a child’s cognitive abilities, impacting their educational future [152]. Some authors have proposed that the socioeconomic differences observed are due to more educated parents, who are better informed about protective or deleterious factors and can ensure optimal child development as early as prenatal life [153,154]. The time parents spend with their children, in the form of physical or cultural activities with either or both parents, is relevant, but so is their overall exposure to discussion and social interaction [155,156]. In addition, it was found that more positive feelings of family dynamics were associated with large hippocampal CA1 and CA2/3 volumes, regardless of age and controlling for socioeconomic status [144].

In the United States, there is an enrichment program called Head Start. It was launched in 1965 to prepare low-income children (between three and five years old) for kindergarten by providing school, social, health, and nutritional support. This program also incorporates parents. The results of this program present evidence in favor [157] and against [158] the program. The Head Start Impact Study (HSIS) is a randomized controlled trial that studied 4442 children aged three-to-four years through the third grade, and the results were recently published. The authors found that the Head Start program had positive short-term effects on several cognitive assessments but did not affect the social–emotional level [159].

The above findings highlight the potential of EE as an encouraging, non-invasive strategy to prevent deficits in cognitive function induced by PNS. Moreover, accumulating evidence suggests that the gut microbiome might be the key connection between positive stimulus and cognitive function. It was observed that changes in gut microbiota improved cognitive alteration induced by different noxas [160,161].

### 3.3. Exercise as a Promissory Approach to Improving the Effects of EE

Nowadays, it is completely accepted that physical activity is an effective way to increase cognitive and emotional health in many psychiatric conditions. In particular, several studies have demonstrated that physical activity significantly improved brain function, counteracting the negative effects of aging on cognitive performance and reducing the risk of dementia [162]. Interestingly, it was suggested that physical activity in humans correlates with the gut microbiome, which could prevent the incidence and development of Alzheimer’s disease [163]. However, there are no studies that have analyzed the effects of physical activity on cognitive deficits induced by PNS.

In rodents, the effects of physical activity or EE on behavior, memory, neurobiology, and underlying molecular biology were investigated individually but not in combination. It was found in rodents that short-term EE improved age-related cognitive decline and anxiety-like behavior without altering hippocampal gene expression [164]. In contrast, physical activity had a detrimental effect on both cognitive- and affective-like behaviors at a young age but not at a middle or late middle age, despite altering hippocampal gene expression [164]. Rodent models of aerobic exercise either use involuntary treadmill-running or voluntary wheel-running paradigms. Using these models, it was observed that voluntary exercise had beneficial effects and, on the contrary, involuntary exercise upregulated the subject’s stress response, leading to a diminished neuroprotective effect [165,166].

The effects of EE with or without a running wheel were studied, and a memory improvement due solely to the cognitive and sensory stimulation produced by EE was observed with no additional effects of exercise [167]. However, the effects of physical activity alone or in combination with EE in models of early life stress have not been exhaustively analyzed. Campbell et al. (2022) [168] performed an interesting review of papers investigating the ability of physical activity to reverse or mitigate the negative effects of early life stress on BDNF expression. In general, it was found that rats exposed to early life stress showed increased anxiety- and depressive-like phenotypes, and these phenotypes were ameliorated by exercise [168]. This positive effect was found mainly in male rats exposed to MS, with missing information on female rodent outcomes and sex differences. However, there was some evidence that stress and exercise differentially affect both sexes. Thus, exercise improved anxiety-like behaviors in male rats exposed to MS but aggravated these behaviors in females exposed to MS. It is important to note that the timing and duration of exercise exposure as a treatment intervention has not been standardized. However, results have indicated that aerobic exercise may be a valuable treatment mechanism for neurodevelopmental, autoimmune, and psychological disorders [168]. The therapeutic benefits of exercise on disease underline a promising future for exercise intervention models.

The effects of EE are built on a complex interaction between multiple environmental factors rather than a single driver [169]. However, all EE treatments are likely to result in increased exercise as a result of the enhanced physical activity from the use of items added to the enclosures. This may explain why the inclusion of the running wheel results in minimal variation in the observed benefits of EE exposure. In addition to the type of exercise, the duration and age of exposure may play a role in its effectiveness [170].

Future studies should investigate sensitive periods of exercise exposure as well as the sufficient duration of exposure for epigenetic and behavioral outcomes, as this will be needed to develop standardized practices in the exercise intervention field.

**Table 2:** Beneficial effects of environmental enrichment after perinatal stress exposure.

Stress Type	Period of Stress Exposure	Period of Enviromental Enrichment	Behavioral Test Used	Age at Behavioral Test	Effects Observed after EE Exposure	Reference
restraint	GD13–GD19	P11–P30	Morris water maze test	P45	↓ Latency time in finding the platform	[171]
					↓ Total swin distance	
					↓ Linear search strategy	
bystander	GD10–GD17	P22–P52	Morris water maze test	P52	Non-beneficial impact on spatial memory and learning	[172]
broadband traffic noise	GD15–GD21	P21–P51	Morris water maze test	P22–P51	↓ Time finding the platform	[128]
					↓ Distance travelled	
restraint	GD12–GD18	P28–P49	Morris water maze test	P57–P64	Foraging enviroment: ↑ time in target quadrant in males	[173]
maternal separation	P2–P20	P21–P54	8-arm radial maze win-shift	P38–P56	↓ Overall errors in both sexes	[174]
maternal separation	P2–P21	P21–P65	Morris water maze test	P92	↑ Time spent in target quadrant (EE vs. NE, all groups) (MS had no effect.)	[175]
maternal separation	P1–P10	P21–P77	Morris water maze test	P21–P77	↑ Time in the target quadrant	[117]
			Novel object recognition		↑ Exploration time of novel object	
maternal separation	P1–P21	P23–P65	Morris water maze test	P70–76	↑ Time spent in target quadrant and frequency of entries	[139]
			Novel object recognition		MS shows non difference vs. non MS.	
maternal separation	P2–P15	P21–P50	Morris water maze test	P52–P70	↑ Time spent in target quadrant (males)	[176]
maternal separation	P1–P21	P22–P34	Morris water maze test	P35–P39	EE eversed all parameters to control group levels.	[177]
			Novel object recognition		MS did not induce recognition memory impairment.	
maternal separation	P1–P21	P21–P51	Morris water maze test	P52–P58	MS increased memory, without effects of EE.	[178]

Abbreviations used: ↓: decrease, ↑: increase.

## 4. Cognitive Deficits and Melatonin Treatment

Melatonin (MT), also known as N-acetyl-5-methoxytryptamine, is a neurohormone primarily synthesized in the pineal gland. Lerner, in 1958, was the first to isolate melatonin from a bovine pineal gland extract as a natural skin-lightening substance [179]. Afterward, an expanding list of functions were discovered, indicating that MT is a hormone with pleiotropic biological functions. Its classical function is a circadian rhythm regulator. MT production is confined to the dark phase of the night, synchronizing the physiological and behavioral relationship of an organism to the external daily and seasonal light/dark environment [180]. Moreover, MT is essential in the regulation of energy metabolism, including body weight, insulin sensitivity, and glucose tolerance [181]. In addition, MT plays a complex role as a modulator of the immune system, restoring immunity in immunosuppression and exhibiting anti-inflammatory properties during inflammation [182]. According to its chemical characteristics, MT is an amphiphilic tryptophan-derived indoleamine with antioxidant properties, which is of importance for mitochondrial functions [180]. The presence of MT has been described in the gastrointestinal tract [183], where it is synthesized independently from pineal production after L-tryptophan is incorporated into the gut from the diet [184]. MT is highly enriched in the digestive tract, especially in the intestine, in higher concentrations than in circulation [185]. After synthesis, MT produces local and systemic effects via its release into the bloodstream or by remaining in the gut lumen and exerting numerous functions [186]. Concerning gut microbiota, there is a complex interrelationship between MT and intestinal bacteria in maintaining homeostasis: bacteria modulate the synthesis of MT in the gut and MT can regulate the composition of the microbiota. In general, it has been observed that MT has increased the richness and diversity of intestinal microbiota, restored Firmicutes/Bacteroidetes ratio, reduced the amount of certain harmful bacterial genera such as *Proteobacteria Desulfovibrio*, *Peptococcaceae*, and *Lachnospiraceae* and increased the abundance of beneficial genera such as *Bifidobacterium* and *Lactobacillus* [186,187]. Accumulating evidence shows that MT can modulate the abundance of the gut bacterial population in normal circumstances, but does so especially in various pathological states [188].

Since MT has direct access to the central nervous system, it can regulate general and specific aspects of neuronal functions, acting as a neuroprotective hormone [180,189].

### 4.1. Animal Studies

MT has been described as preventing the decrease in adult hippocampal neurogenesis and the deterioration of cognitive abilities after irradiation [190] and improving cognitive memory in a rat model of post-traumatic stress disorder [191]. Concerning pregnancy, although the circadian rhythm is modulated through MT and appears to be essential for a successful pregnancy, experimental findings have indicated that MT administration in pregnant animals provokes decreased birth weight [192], altered circadian rhythm [193], and mortality [194]. However, it has been observed that maternal administration of MT in rats has prevented the decrease in cognitive abilities in offspring related to the loss of hippocampal neurons caused by prenatal irradiation [195], had a neuroprotective effect in LPS-induced brain damage in mice [196], ameliorated oxidative stress, and promoted normal brain structure and function in an ovine model of fetal growth restriction [197].

As described above, stress exposure during pregnancy affects the normal functioning of the HPA axis, immune regulation, and the structure of the gut microbiome in the mother and affects fetal and postnatal offspring neurodevelopment, behavior, and immunity. In a PS study on rats, it was shown that treatment with the MT analog Piromelatine attenuated the high anxiety level, reversed the stress-induced increase in plasma corticosterone levels in both sexes, and decreased the increment of hippocampal–corticosteroid receptor levels observed in males [198]. Another study on Piromelatine treatment in rats found a beneficial effect on the PS-induced alteration of associative memory in both male and female offspring. However, impairment of PS-induced, hippocampus-dependent spatial memory was reversed only in PS-affected males and not females [199]. Moreover, MT treatment prevented hippocampal damage induced by MS in infant rats [200].

### 4.2. Clinical Evidence

The relationship between physiological levels of MT and cognitive function has been studied in an elderly population. A significant association between higher MT physiological levels and a lesser prevalence of cognitive impairment was found [201]. A systematic review of clinical trials assessed for the effects of MT treatment on cognitive function in patients with Alzheimer’s disease suggested that daytime MT administration may be effective for improving cognitive function in patients with a mild to moderate pathology [202]. In patients with mild cognitive impairment syndrome, it was found that MT treatment improved performance [203]. In addition, animal and human studies have found that MT administration exerted an anxiolytic effect [204]. Interestingly, a potential benefit of MT has been found in its prevention of a delirious state in intensive care unit patients after organophosphorus compound poisoning [205], severe sepsis, and septic shock [206], as well as in an elderly patient with severe dementia [207] and in a healthy male patient after a road traffic accident [208].

Concerning pregnancy, a recent review of 15 clinical studies on the use of MT for different clinical purposes during pregnancy and lactation did not suggest adverse maternal or fetal events related to MT administration [209]. However, the authors pointed out a need for robust clinical studies on pregnant and lactating populations.

## 5. Conclusions

**Figure 2:**
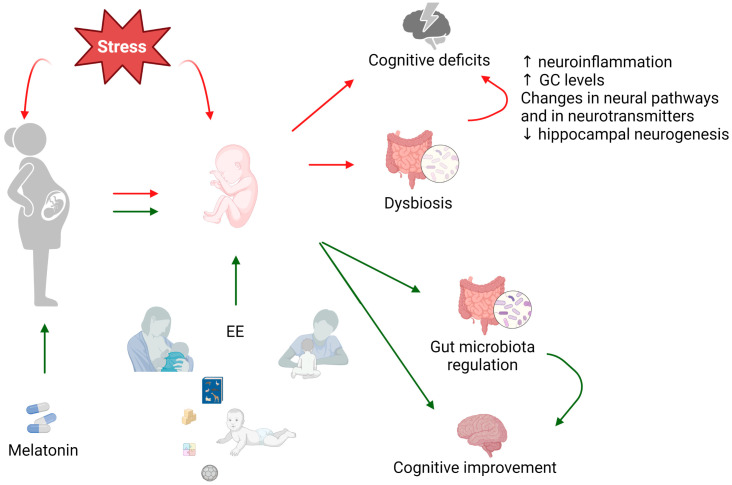
Perinatal stress and cognitive deficits. The period around birth is a time of significant vulnerability and may be influenced by internal and external risk factors that affect fetal and infant development. Direct stress exposure in the offspring or indirect exposure due to changes in the mother’s physiology might induce microbiota dysbiosis, leading to cognitive deficits through different pathways. Environmental enrichment, through physical, sensory, cognitive, and social stimulation, could revert the effects of stress exposure on cognitive performance. Alternatively, the administration of melatonin to the mother could avoid the deleterious effects induced by stress exposure. Red arrows indicate the deleterious effects of stress exposure. Green arrows indicate the positive effects of melatonin treatment and environmental enrichment. The figure was created with the BioRender.com.

In this review, we showed several lines of evidence indicating that PNS can induce cognitive deficits via different pathways, and we discussed potential early treatments—EE exposure and MT administration—for preventing the deleterious effect of perinatal stress exposure (Figure 2). Nowadays, the interaction between gut microbiota and the brain is a topic of great interest. Several studies have suggested that alterations in gut microbiota induce an impairment in cognitive abilities in several pathological conditions. Although experimental and clinical findings point to the outstanding role of the gut–brain axis in the cognitive deficits induced by PNS exposure, there is no direct evidence linking changes in microbiota with its consequences on cognitive performance. While there are a few reports that have indicated that EE and MT treatments are able to improve the cognitive deficits induced by PNS, the data showed in this review encourage the realization of studies that investigate the beneficial role of MT administration and EE exposure. Taking into account the multiple articles describing the influence of sex on different responses, these future studies should be performed on males and females. On the other hand, direct evidence of the restoration of microbiota as a primary mechanism behind the beneficial effects of this treatment has not been fully demonstrated and should be explored in future studies, including on prebiotic and probiotic administration.

## Data Availability

No new data were created or analyzed in this study. Data sharing is not applicable to this article.
